# Does pre-COVID impulsive behaviour predict adherence to hygiene and social distancing measures in youths following the COVID-19 pandemic onset? Evidence from a South African longitudinal study.

**DOI:** 10.1186/s12889-023-15310-w

**Published:** 2023-03-20

**Authors:** Katharina Haag, Stefani Du Toit, Nace Mikus, Sarah Skeen, Kathryn Steventon Roberts, Marguerite Marlow, Vuyolwethu Notholi, Akhona Sambudla, Yeukai Chideya, Lorraine Sherr, Mark Tomlinson

**Affiliations:** 1 Institute for Global Health, University College London, London, UK; 2 Present affiliation: Department of Child Health and Development, Norwegian Institute for Public Health, Oslo, Norway; 3 Institute for Life Course Health Research, Department of Global Health, Stellenbosch University, Cape Town, South Africa; 4 Department of Cognition, Emotion, and Methods in Psychology, Faculty of Psychology, University of Vienna, Vienna, Austria; 5 Interacting Minds Centre, Aarhus University, Aarhus, Denmark; 6 Amsterdam Institute for Social Science Research, Faculty of Social and Behavioural Sciences, University of Amsterdam, Amsterdam, Netherlands; 7 School of Nursing and Midwifery, Queens University, Belfast, UK

**Keywords:** COVID, Impulsivity, Adolescence, LMIC, Longitudinal

## Abstract

**Background:**

Engagement in protective behaviours relating to the COVID-19 pandemic has been proposed to be key to infection control. This is particularly the case for youths as key drivers of infections. A range of factors influencing adherence have been identified, including impulsivity and risk taking. We assessed the association between pre-COVID impulsivity levels and engagement in preventative measures during the COVID-19 pandemic in a longitudinal South African sample, in order to inform future pandemic planning.

**Methods:**

Data were collected from *N* = 214 youths (mean age at baseline: *M* = 17.81 (*SD* = .71), 55.6% female) living in a South African peri-urban settlement characterised by high poverty and deprivation. Baseline assessments were taken in 2018/19 and the COVID follow-up was conducted in June–October 2020 via remote data collection. Impulsivity was assessed using the Balloon Analogue Task (BART), while hygiene and social distancing behaviours were captured through self-report. Stepwise hierarchical regression analyses were performed to estimate effects of impulsivity on measure adherence.

**Results:**

Self-rated engagement in hygiene behaviours was high (67.1–86.1% “most of the time”, except for “coughing/sneezing into one’s elbow” at 33.3%), while engagement in social distancing behaviours varied (22.4–57.8% “most of the time”). Higher impulsivity predicted lower levels of hygiene (*β* = .14, *p* = .041) but not social distancing behaviours (*β* = −.02, *p* = .82). This association was retained when controlling for a range of demographic and COVID-related factors (*β* = .14, *p* = .047) and was slightly reduced when including the effects of a life-skills interventions on hygiene behaviour (*β* = −.13, *p* = .073).

**Conclusions:**

Our data indicate that impulsivity may predict adolescent engagement in hygiene behaviours post COVID-19 pandemic onset in a high risk, sub-Saharan African setting, albeit with a small effect size. For future pandemics, it is important to understand predictors of engagement, particularly in the context of adversity, where adherence may be challenging. Limitations include a small sample size and potential measure shortcomings.

**Supplementary Information:**

The online version contains supplementary material available at 10.1186/s12889-023-15310-w.

## Background

Following the outbreak of the COVID-19 pandemic, social distancing and hygiene practices were suggested as key measures for limiting infections and deaths, and to prevent health systems from becoming overwhelmed [1]. Recommended social distancing practices included an avoidance of close physical contact to individuals outside the household [2], while hygiene practices comprised hand and respiratory hygiene measures (e.g., hand sanitising, mask wearing). Early evidence from initial lockdowns (approximately March–June 2020) across sub-Saharan Africa indicates moderate to high adherence levels to protective behaviours [3, 4], but also that these decline over time [5]. Factors predicting adherence were demographic (including age, education, poverty, living situation), psychological (perceived COVID-risk and adherence barriers, self-efficacy), and/or COVID-related (e.g., knowledge, dissatisfaction with government measures) in nature [5–7]. However, costs of engagement in such measures may be particularly high for youths, who have a lower risk of severe illness, but for whom social distancing in particular may require forgoing income or social opportunities [8]. In accordance, it was proposed from mid-2020 and onwards that young adults may be key drivers of COVID-19 infections across the globe, including in African settings [9, 10]. Even though the world is reopening, the need to prevent COVID-19 remains. Therefore, it is important to understand predictors of engagement in young people living in Sub-Sharan Africa, particularly in contexts of poverty or high population density, where adherence may be challenging [11, 12]. This information can also be valuable for future pandemic planning.

Impulsive individuals have been shown to struggle with anticipating future consequences of behaviours, and to be more likely to act prematurely, take risks and be easily distracted [13]. Since COVID-related hygiene and social distancing measures commonly require deviations from habitual and automatic responses, impulsive individuals may struggle to maintain them [14, 15]. In HICs, impulsivity, alongside factors such as opportunities to break rules, has been linked to poorer engagement in COVID-related protective behaviours both cross-sectionally [16, 17] and longitudinally [18]. Furthermore, in a Turkish sample, impulsivity was associated with both lower H1N1- and COVID-related hygiene behaviours [19, 20]. Similarly, higher impulsivity was linked to poorer health-behaviours and higher COVID infection rates during the first Mexican lockdown [14]. There is also a range of indirect evidence, suggesting lower hygiene behaviours in individuals with conditions associated with increased impulsivity, such as attention deficit hyperactivity disorder [21] and anti-social personality disorder [22]. However, there is a lack of longitudinal data and many of the above-mentioned studies use questionnaire rather than behavioural measures of impulsivity and risk-taking. Longitudinal data are valuable as they allow investigating whether population baseline levels of impulsivity/risk-taking predict pandemic behaviours, and whether intervention is warranted for future pandemic planning. Furthermore, previous studies were predominantly conducted in adults, with youths having been proposed to engage more frequently in risky behaviours [23].

Within sub-Saharan Africa, impulsivity has often been studied in the context of youths’ health risk behaviours, such as risky sex, gambling, or substance use [24, 25]. To the best of our knowledge, impulsivity has not been explored as a potential risk factor for poor COVID measure compliance but may an important target behaviour in future pandemic planning and readiness. We also had a secondary aim of utilising the current data to derive recommendations for public health communication and implementation of protective measures for future pandemics. We utilised data from a longitudinal study conducted from birth until current ages 19–21 years in an impoverished neighbourhood of Cape Town, South Africa, to gain a first impression of such associations. Before the current COVID-assessments, data were most recently collected following a randomised control trial of a life skills intervention specifically designed for adolescents living in adverse contexts and aimed at teaching youths' skills such as future planning and reflection on their behaviours.

We hypothesized that youths’ risk taking in a behavioural task measured before onset of the COVID-19 pandemic (i.e., their “baseline” behaviours) would be associated with lower engagement in hygiene and social distancing behaviours post COVID-19 pandemic onset. We controlled for and performed secondary analyses on the effects of a pre-COVID adolescent life skills intervention on any relationships found between impulsivity and protective behaviour engagement. With the life skills intervention having previously been found to affect impulsivity in young men in particular, we also expected that it may serve as a mediator of the found associations.

## Methods

### Study design and setting

**Fig. 1:**
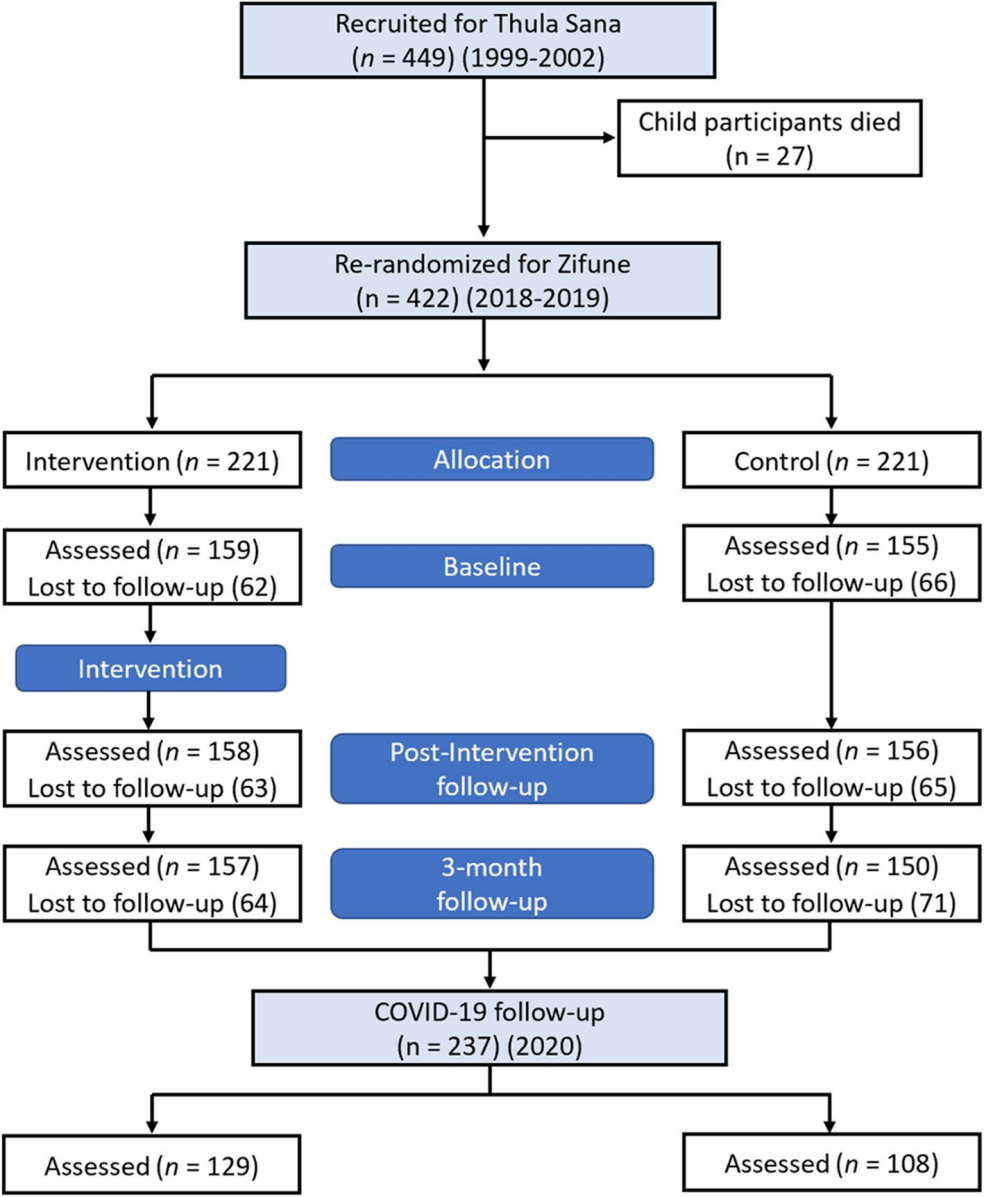
CONSORT Flow Diagram for Cohort Studies

The sample was drawn from a longitudinal intervention study conducted in peri-urban Khayelitsha, South Africa, which followed children and their families from before birth until current age (19–21 years; see Fig. 1 for a CONSORT flowchart of assessments). From the antenatal period until 6 months after birth, expectant mothers received either a parenting intervention (‘Thula Sana’; *n* = 220) aimed at improving parenting skills and attachment, or maternal services as usual (control group, *n* = 229). All mothers in the community who were eligible for study participation were invited, and group assignment was randomized. Families were followed up several times over the first 18 months of the child’s life [26] and again at 13 years of child age [27]. No effects of the early intervention on adolescent outcomes were identified [27] and we subsequently do not control for receipt of this intervention in the current study. The youths then underwent a second intervention (‘Zifune’; for details, see below), aimed at teaching life skills to improve pro-sociality and reduce violence behaviours, at ages 16–19 years (*n* = 319; re-randomized based on early intervention group allocation) (Skeen S, Du Toit S, Marlow M, Stewart J, Rabie S, Melendez-Torrez GJ, et al: Zifune: Does a second wave intervention delivered to former recipients of an early mother-infant attachment intervention reduce interpersonal violence during adolescence? A re-randomized controlled trial, in preparation). Data collection took place in 2018/2019, at three time-points: pre-intervention, directly post-interventions (*n* = 314) and at a 3-month follow-up (*n* = 307). At the post-intervention assessment, participants completed several behavioural tasks to investigate whether the intervention had led to any changes in risk taking and moral behaviours (*n* = 280).

### Life skills intervention

The Zifune life skills intervention was developed for use with youths living in high adversity contexts in low- and middle-income countries (LMIC) (Skeen S, Du Toit S, Marlow M, Stewart J, Rabie S, Melendez-Torrez GJ, et al: Zifune: Does a second wave intervention delivered to former recipients of an early mother-infant attachment intervention reduce interpersonal violence during adolescence? A re-randomized controlled trial, in preparation). An adolescent advisory board provided feedback to ensure applicability and acceptability of its contents. The intervention utilises a collaborative approach, incorporates principles of cognitive behaviour therapy, and employs creative and fun methods to allow youths to reflect on their relationships and behaviours and to devise future plans. Eight group-based sessions for groups of approximately 20 youths each were provided by trained facilitators from the local community. Sessions covered six main themes: vision for the future, time management, financial planning, mindfulness, risk-taking behaviour and interpersonal violence, with sessions about long-term planning and risk-taking behaviours in particular potentially affecting impulsivity levels. An intervention facilitator remained in regular contact with and provided support to the youths throughout the course of the study via phone calls.

### Data collection during the COVID-19 pandemic

Following the outbreak of the COVID-19 pandemic, South Africa went into a strict lockdown in March 2020. Brief telephonic interviews were conducted with participants (*n* = 237) in June to October 2020 through remote-working data collectors. During this time, South Africa’s first large case wave took place (July–August 2020), followed by a strong decline in cases. Participants were assessed on a range of COVID-related variables, including social distancing and hygiene behaviours, household food security, mental health, and schooling outcomes. We utilize data from those who took part in the behavioural tasks at the post-intervention assessment of the Zifune study and completed the COVID-related questionnaire after the pandemic outbreak (*n* = 214).

#### Consent and procedure

All participants provided written consent at each wave of the data collection. Assessments were conducted in the participants’ language of choice, predominantly isiXhosa. All data were collected by trained and supervised data collectors, with at least a high school diploma and with prior experience in working with vulnerable populations. For the current phase of the study, ethical approval was obtained from the Health Research Ethics Committee (HREC) from Stellenbosch University (Ref: N17/10/094).

#### Measures

##### Demographic variables

Information on the gender, age, level of education (utilised in the form of a “correct grade for age” variable), housing (formal vs informal housing); number of household members the individual was living with during the COVID-19 pandemic, HIV status, and household receipt of any form of government-provided cash grants was collected.

##### Impulsivity/risk taking - balloon analogue risk task (BART)

The BART [28] is a naturalistic computer task measuring impulsive and risk-taking behaviours. Participants are presented with a balloon, which they can enlarge in a step-wise fashion by pressing a button. Each pump increases the reward pay-off that the participant receives, but also the chance of the balloon popping, which leads to no rewards for the trial. Participants have the choice to step away after each button press, and collect the already accrued rewards for the trial, or to keep pumping. In the current study, all participants were asked to complete 30 trials. They were told that one trial would be chosen at random in the end, for which they would receive the earned monetary reward. To even out expectations, all participants observed 12 balloons being inflated to their bursting point before commencing the task. The bursting point was set to be identical in each trial between participants. The overall number of pumps was used as a predictor of interest, with a higher number of pumps reflecting higher risk taking.

##### Hygiene Behaviours

The extent to which participants engaged in each of four hygiene behaviours (hand washing, hand sanitising, coughing/sneezing into one’s elbow, and wearing a face mask) during the past week was measured on a scale from 0 “never” to 3 “most of the time” for each item (see Additional file 1: Appendix 1 for full item list and rating scale). A total score (0–12) was calculated. An exploratory factor analysis revealed that the items did not load well onto a single underlying factor, potentially due to participants picking and choosing certain behaviours or adhering less stringently to measures as the pandemic situation in South Africa relaxed towards September/October 2020. As a result, we decided to investigate the total score, reflecting the overall extent of hygiene behaviours each participant engaged in, but also analysed the four behaviours separately to see whether any effects found were driven by high scores on particular items.

##### Social distancing Behaviours

The extent to which participants engaged in five social distancing practices during the past week was assessed: keeping a 1–2 m distance, and avoiding public transport, going to the shops/pharmacy, public spaces and going for a walk in the neighbourhood. Items were rated from 0 “never” to 3 “most of the time” and summed up into a total score (0–15). Exploratory factor analysis suggested that the latter three items loaded onto a potential “avoidance of public outings” factor, though individual item loadings were small. Therefore, we chose to investigate the total score, indexing the extent of overall social distancing behaviours, and to additionally explore single-item effects.

##### Potential confounders

We added age and sex to the analyses, since risk behaviours in the BART have been shown to be influenced by both factors. We furthermore controlled for education (being in the correct grade for age) and timing of the assessment, since the COVID situation changed substantially in South Africa throughout our data collection, from the first case wave in June/July 2020 to level 1 restrictions in September 2020. In terms of COVID-related factors that could have influenced participants’ abilities to engage in hygiene and social distancing behaviours, we adjusted our analyses for household food security as a measure of deprivation (Household Food Insecurity Access Scale (HFIAS, [29]), and the number of individuals living in the participant’s household, which could have desensitized participants to being around large groups of people, or heightened worries and subsequent measure engagement, especially in multi-generational households. Finally, we controlled for receipt of the life skill intervention at ages 16–19 years, as it was found to influence risk taking in males particularly (Mikus N, Skeen, S, Stewart J, Marlow M, DuToit S, Rabie S, Mendelez Torres GJ, et al: Psychosocial intervention improved self-control in adolescents, in preparation).

#### Statistical analysis

Analyses were conducted using StataSE 16 and R 4.1.1 In a first step, we investigated descriptive characteristics of the sample and compared it to participants who had completed the BART impulsivity measure and were not included in the COVID follow-up on relevant demographic factors, using t-tests and χ^2^ tests as appropriate. We then performed Pearson’s correlation analyses between the key variables. Finally, based on findings from the correlation analyses, a hierarchical linear regression analysis was performed, with hygiene behaviours as the key outcome. In the first step, impulsivity was added as a predictor, with higher pumps on the BART indexing higher impulsivity/ risk taking. Secondly, the demographic factors of age, sex and correct class for age were included. In a third step, COVID-related factors (food security, number of people living in the household, time to level 1 restrictions) were added to the model. In a last step, receipt of the life skills intervention was added, to see whether any effects found may be explained by exposure to its contents. Finally, since the intervention was found to affect BART-measured impulsivity in a previous study (Mikus N, Skeen, S, Stewart J, Marlow M, DuToit S, Rabie S, Mendelez Torres GJ, et al: Psychosocial intervention improved self-control in adolescents, in preparation) and showed close to significant predictions of hygiene behaviours (*β* = .09, *p* = .190) in the current study, we conducted secondary exploratory causal mediation analyses, using the “mediation” package in R 4.1.1 [30]. The aim was to investigate whether the life skills intervention may be able to buffer potential associations between higher impulsivity and lower protective behaviour engagement. For this, we explored whether any indirect effects of exposure to the life skills intervention on hygiene behaviours through impulsivity would be found. However, we acknowledge limited power due to a small sample size.

## Results

### Descriptive information

**Fig. 2:**
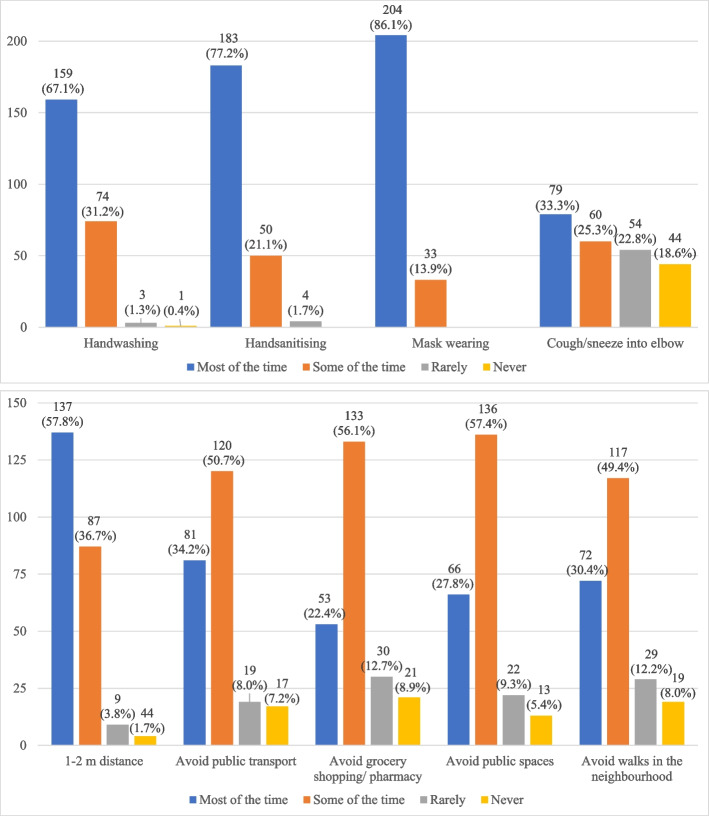
Frequency of individual hygiene and social distancing behaviours in the week before the COVID-19 pandemic follow-up

The mean age of the included sample was *M* = 17.81 (*SD* = .71; range: 16–20) years at the time of the BART data collection; and *M* = 19.52 (*SD* = .60; range 19–21) years at the COVID follow-up. 55.6% (*N* = 119) of participants were female. Further descriptive information on the sample is provided in Table 1. The average number of pumps on the BART across the 30 trials was *M* = 21.13 (*SD* = 6.68; range: 6.82–37.17). Participant responses to the hygiene and social distancing behaviour items are illustrated in Fig. 2. For hygiene behaviours, the mode answers showed participants adhered to them “most of the time”, except for “coughing and sneezing into one’s elbow”, which only 33.3% of participants engaged in “most of the time” and 40.7% never or rarely engaged in. Twenty-two percent (*n* = 47) showed adherence to all four hygiene behaviours “most of the time”, with average total scores lying at *M* = 10.02 (*SD* = 1.55, range: 6–12). For all social distancing behaviours, the mode was “some of the time”, except for keeping a 1–2-m distance, which a majority of participants complied with “most of the time”. 4.2% (*n* = 9) participants engaged in all social distancing behaviours “most of the time” over the past 7 days; the average total score was *M* = 10.62 (*SD* = 2.41, range: 3–15).

**Table 1:** Baseline and COVID-19 pandemic sample characteristics (*N* = 214)

Baseline sample	Statistic
Mean Age in Years	*M* = 17.81 (*SD* = .71)
Sex (1 = female)	119 (55.6%)
Living in Informal Housing	21 (9.9%)
HIV Positive	9 (4.6%)
Correct Class for Age	97 (45.5%)
Household Cash Grant Receipt	117 (54.9%)
**COVID-19 pandemic sample**	
Mean Age in Years	*M* = 19.53, (*SD* = .60)
Number of People in Household	*M* = 3.79, (*SD* = 1.91)
Food Insecurity Score (0–27)	*M* = 7.68 (*SD* = 5.95; range 0–22)

For assessment of baseline characteristics, data from the 3-months follow-up were used, since this was when the BART was completed

Comparing the sub-samples that were and were not followed up as part of the COVID-19 pandemic assessment (loss to follow-up: 24.5%), we found that females (55.6% versus 44.4%, *χ*^*2*^ = 4.37, *p* = .037) and those receiving the life skills intervention (56.1% versus 43.9%, *χ*^*2*^ = 5.62, *p* = .018) were more likely to have taken part in the follow-up study. Those followed up had somewhat lower average BART pumps, indexing lower impulsivity (*M*_excluded_ *=* 22.88, *SD* = 7.67, *M*_included_ = 21.13, *SD* = 6.68; *p* = .007). No group differences were found in terms of mean age, if the participants lived in formal versus informal housing and were in the correct school class for their age, and whether the household the adolescent lived in received any social grants (all *p* > 0.55).

### Correlation analyses between protective behaviours, impulsivity, and potential confounders

Correlation analyses (Table 2) indicated that higher impulsivity (indexed by the number of BART pumps) was negatively associated at a small effect size (*r* = −.14, *p* = .041) with the extent of hygiene behaviours, but not with social distancing behaviours. It was also negatively linked with being of female sex (*r* = −.16, *p* = .021) and having received the life skills intervention (*r* = −.15, *p* = .026). Hygiene and social distancing behaviours were positively correlated at a small effect size (*r* = .21, *p* = .002). The extent of hygiene behaviours was also marginally negatively associated with age (*r* = −.12, *p* = .082) and time to level 1 COVID restrictions (*r* = −.13, *p* = .057) and positively with the number of household members (*r* = .11, *p* = .097). The extent of social distancing behaviours was positively associated with female sex (*r* = .22, *p* = .001). Since the hygiene and social distancing items did not load onto the same underlying factors, we also conducted analyses using single items (Additional file 2: Appendix 2). Associations between impulsivity as measured by the BART and individual hygiene behaviours followed the same direction as for the total score for three out of the four hygiene items (except for mask-wearing), though at somewhat smaller effect sizes. For the social distancing items, associations were small/non-significant and in mixed directions, reflecting the overall lack of relationship found between impulsivity (BART pumps) and the total social distancing behaviour score. Resultingly, we report the results of multiple regression analysis only with the total hygiene behaviour score as an outcome. However, equivalent analyses for social distancing behaviours are reported in Additional file 3: Appendix 3.

**Table 2:** Bivariate correlation between impulsivity, hygiene and social distancing behaviours, and potential confounders

	1.	2.	3.	4	5.	6.	7.	8.	9.
**1. Impulsivity (BART pumps)**	**–**								
**2. Hygiene Behaviours Total Score**	−.14*	–							
**3. Social Distancing Behaviour Total Score**	−.02	.21*	–						
**4. Age**	−.03	−.12	.06	–					
**5. Sex (1 = female)**	−.16*	.04	.22*	.09	–				
**6. Correct class for age**	−.09	−.05	−.07	.10	−.08	–			
**7. Intervention (1 = Received)**	−.15*	.11	.11	.04	.20	−.05	–		
**8. Time to Level 1 Restrictions**	.03	−.13	−.08	.03	.02	−.04	.03	–	
**9. Number of Household Members**	−.09	.11	−.02	.03	.06	.03	.03	−.08	–
**10. COVID Food Security**	.06	−.03	−.08	.01	−.02	.12	−.04	.13	.01

### Multivariable models predicting hygiene behaviours

In the first step, higher impulsivity (i.e., a higher number of BART pumps) negatively predicted the total amount of hygiene behaviours at a small effect size (*β* = −.14, *p* = .045), explaining 1.5% of their variance (Table 3). This effect was retained once the demographic confounders were included (*β* = .15; *p* = .035), though the overall model (*F* (4,208) = 2.01, *p* = .095) was not significant at level *p* < .05. Once COVID-related variables were controlled for, the model regained significance (*F* (7,205) = 2.12, *p* = .04), and impulsivity continued to predict the total hygiene behaviour score at a small effect size (*β* = .14, *p* = .047). Since the life skills intervention was by itself found to predict lower impulsivity in males, we examined how the effect of impulsivity on hygiene behaviours changed when including the life skills intervention as a predictor variable. We found that the degree to which impulsivity predicted hygiene behaviours was slightly reduced (*β* = .13, *p* = .073), when the receipt of the life skills intervention variable was included in the model.

**Table 3:** *Step-wise regression model predicting COVID hygiene behaviours during the past 7 days*

	B	95% CI	β	p
**Step 1**				
Impulsivity (BART pumps)	−.03	−.06; −.00	−.14	.041
*F, p, adjusted R*^*2*^	*4.21; .041; .015*			
**Step 2**				
Impulsivity (BART pumps)	−.03	−.07; .00	−.15	.035
Sex (1 = female)	.05	−.38; .47	.01	.830
Age	−.30	−.65; .05	−.12	.094
Correct Class for Age	−.16	−.58; .26	−.05	.461
*F, p, adjusted R*^*2*^	*2.01, .095, .019*			
**Step 3**				
Impulsivity (BART pumps)	−.03	−.06; .00	−.14	.045
Sex (1 = female)	.02	−.41; .45	.01	.927
Age	−.28	−.63; .07	−.11	.113
Correct Class for Age	−.27	−.71; .16	−.09	.214
Food Insecurity	.00	−.04; .03	.00	.992
Number of Household Members	.09	−.02; .21	.12	.097
Time to Level 1 Restrictions	−.01	−.02; .00	−.13	.066
*F, p, adjusted R*^*2*^	*2.12; .043; .036*			
**Step 4**				
Impulsivity (BART pumps)	−.03	−.06; .00	−.13	.073
Sex (1 = female)	.00	−.43; .43	.00	.999
Age	−.29	−.63; .06	−.11	.104
Correct Class for Age	−.27	−.71; .15	−.08	.204
Food Insecurity	.00	−.03; .04	.00	.955
Number of Household Members	.09	−.02; .20	.11	.103
Time to Level 1 Restrictions	−.01	−.02; .00	−.13	.058
Intervention (1 = yes)	.28	−.13; .71	.09	.180
*F, p, adjusted R*^*2*^	*2.09; .038; .039*			

*B* Unstandardized regression coefficient, *CI* Confidence interval, *β* Standardized regression coefficient

### Mediation analysis for the life skills intervention

This prompted us to explore whether the intervention itself had an effect on hygiene behaviours and whether it was mediated by impulsivity. We found an average causal mediation effect (ACME) of .03 (95% Quasi-Bayesian Confidence Interval (QBCI) = [−.00, .10]; *p* = .098), and an average direct effect (ADE) of .18 (95% QBCI = [−.10, .46], *p* = .218). The total estimated effect was .21 (95% QBCI = [−.06, .49], *p* = .135) and the proportion mediated .13 (95% QBCI = [−.99, 1.46], *p* = .221). Effects lay at identical sizes when a moderation by sex was included (ACME = .03, 95% QBCI = [.00, .10], ADE = .18, 95% QBCI = [−.10, .46]).

## Discussion

In a South African sample, we found that pre-COVID impulsivity levels predicted hygiene but not social distancing behaviours following the outbreak of the COVID-19 pandemic at a small but significant effect size. This effect was retained when controlling for a range of demographic variables and factors measured post COVID-19 pandemic onset. When we included the life skills intervention in the model, which aimed to increase prosociality and reduce violence behaviour, the effects of impulsivity were slightly reduced. This suggests that the total effect of impulsivity on hygiene behaviours is partially due to the effects of the intervention on hygiene behaviours. When investigating the effect of the intervention on the main outcome variable, we found some evidence that the life skills intervention itself had a positive (but insignificant) effect on hygiene behaviours, that was mediated by the effects of the intervention on impulsivity with a trend (*p* = 0.09).

This study was not specifically set up to investigate COVID-related changes and was somewhat limited in sample size and resulting analytic power. Thus, findings should not be over-interpreted. However, given that longitudinal data predicting hygiene and social distancing behaviours post COVID-19 pandemic onset and data from LMICs in particular is lacking, it still offers some relevant insights that can be applied to future pandemic planning:

Firstly, in accordance with studies from high income countries (HIC) [15–18], we found that pre-COVID impulsivity predicted lower engagement in hygiene behaviours, though at a small effect size. This supports the notion that challenges such as lack of behavioural inhibition, difficulties to anticipate long-term consequences and altered reward processes may make it more difficult for impulsive individuals to engage in health-related protective behaviours [31, 32]. The BART has been proposed to measure impulsive choice and decision making naturalistically and with a higher objectivity and external validity than questionnaires [28]. While it may be a concern that the BART predominantly examines risk-taking relating to small monetary rewards, it has previously also been linked to health-behaviours, such as smoking and alcohol use [33, 34] and may work similarly for sexual rather than monetary rewards [35], suggesting a degree of generalizability. However, other studies have found relatively low correlations of the BART with multiple facets relating to real-life impulsivity and risk taking [36], which means we may be under-estimating associations. Future studies should aim to investigate more specific underlying processes (e.g., low inhibition versus reward-responsiveness) and potential mediators (e.g., altered risk perceptions) to identify specific behaviours and pathways that can be targeted by interventions. Given the small associations found, it may also be important to investigate specific subgroups that showed high impulsivity during the pandemic and associations of impulsivity/risk-taking during the pandemic with both, baseline impulsivity levels pre-pandemic and measure engagement post pandemic onset.

Second, life skills interventions can reduce impulsivity levels in the general population. The life skills intervention investigated here aimed to teach a range of skills, such as thinking through actions before engaging in them (Skeen S, Du Toit S, Marlow M, Stewart J, Rabie S, Melendez-Torrez GJ, et al: Zifune: Does a second wave intervention delivered to former recipients of an early mother-infant attachment intervention reduce interpersonal violence during adolescence? A re-randomized controlled trial, in preparation) and was found to affect risk taking measures in males in particular (Mikus N, Skeen, S, Stewart J, Marlow M, DuToit S, Rabie S, Mendelez Torres GJ, et al: Psychosocial intervention improved self-control in adolescents, in preparation). Despite not acting as a mediator in our analysis, the trends found suggest that modification of behaviours may be possible and could affect real life outcomes and increase hygiene behaviour adherence. With impulsivity also affecting a range of other key health behaviours [37] and better self-regulation potentially acting as a buffer for healthy development in challenging environments [38] there is a need for public health policies that address its variable levels, ideally through utilising integrative programmes and interventions that focus on its early developmental precursors [38–43].

Third, in accordance with our secondary aim of providing relevant data for future pandemic planning, we found that in a LMIC context characterized by poverty and deprivation, self-reported engagement in hygiene measures was high, but as found in other studies, reduced somewhat as the pandemic situation in South Africa relaxed [5, 44]. With mask wearing being mandated from May 1st, 2020, and the government and NGOs strongly advertising the other three hygiene measures assessed here [2, 45], our study suggests that individuals can both extend and adjust known health behaviours such as hand washing and adopt novel behaviours such as mask wearing if targeted by effective public health messaging. Importantly however, observational evidence indicates that real-life adherence may lie substantially below self-reported levels [46], highlighting potential social desirability and self-selection effects in surveys, and the limitations of self-report. Thus, our study may also be prone to over-reporting engagement. Our results also show that very few individuals consistently engaged in all four recommended hygiene behaviours, mirroring similar findings from South Africa indicating that only 35% of survey respondents followed a high impact set of preventative behaviours [47]. Together, these findings suggest that despite high existing knowledge and awareness of the efficacy of hygiene measures [48], these may ultimately not be adopted consistently. However, part of these findings may also be explained by certain measures being incompatible (e.g., mask wearing and sneezing into one’s elbow), or mask wearing being mandated, which may explain why no associations were found between mask wearing and impulsivity on an individual level.

Of note, self-reported engagement in social distancing behaviours was lower than for hygiene behaviours, potentially due to fewer targeted health messages, different perceived social norms [49], or a lower ability to engage in some of the behaviours (e.g., avoiding going to the shops or using public transport to get to work). Alternatively, it may be that the phrasing of the items (asking for instance how frequently a behaviour was avoided, rather than engaged in) could have affected reported frequencies and thus the validity of the results. Varying compliance with different types of measures has also been established in the Argentinian context [50], with divergent factors predicting engagement in different measures. Overall, there will be a need to better understand and address intersecting vulnerabilities (race, gender, poverty [reflected for instance in high population density, large households, communal water sources], and geopolitics) in sub-Saharan Africa contexts [48, 51, 52] to increase rates of compliance with protective measures in future pandemics.

Fourth, female sex predicted higher engagement in social distancing but not hygiene behaviours. This is of interest, since the former measures were less strongly mandated by official sources and may require higher voluntary engagement. Our findings are in accordance with other studies suggesting lower rates of male engagement in hygiene and social distancing behaviours during the COVID-19 pandemic, but also previous pandemics such as SARS and MERS [53, 54]. One potential explanatory factor is male socialization, which may lead to men being more likely to mask fear, downplay risks and/ or engage in high-risk behaviours [55]. Studies from HIC contexts also suggest that females may have higher individual risk perceptions [56], may be more willing to cooperate with mandated health measures [57] and may be overall more health conscious [58]. Furthermore, while both men and women are employed in keyworker positions, men may be more likely to be the breadwinners for families in the sub-Saharan African context, and thus more likely to struggle to adhere to social distancing (e.g., during transport to work, at the workplace). Future studies will need to further disentangle sex effects, and targeted interventions for males in particular may be required.

Overall, our study highlights the need to apply targeted approaches for both health messaging during a pandemic and future intervention planning. Efforts should be directed towards specific subgroups of individuals that may be at particular risk of not engaging in protective behaviours [54, 55]. Our findings add to a range of studies investigating potential demographic, psychological, personality and structural predictors [59] that can guide such efforts.

The study has several strengths, including the utilization of longitudinal data from a sub-Saharan African, high-risk context. However, it also has several limitations. Firstly, the measures used to assess hygiene and social distancing behaviours had not previously been validated and did not load onto the same underlying factors. The behaviours assessed by the social distancing items may also have been affected by unassessed secondary factors such as the need to use public transport for work. However, the items still capture a range of key behaviours recommended by governmental and non-governmental agencies. Secondly, given that only a subset of youths had completed the behavioural tasks and that it was challenging to reach all participants remotely, the sample size was somewhat low, limiting our analytical power. Thirdly, the follow-up study was designed to get a rapid overview of COVID-related outcomes and questionnaires were kept brief to prevent drop-out in telephonic interviews. Thus, some constructs potentially relevant to the study such as risk perceptions or being unable to socially distance due to workplace conditions were not assessed, which means effects may be over- or underestimated. Fourth, COVID assessments were collected within 1–1.5 years post-intervention assessment. While substantially different in content, participants could have considered the COVID follow-up as another part of the intervention evaluation. This may have made the intervention group more prone to social desirability, self-selection and continuation effects, potentially leading to higher levels of self-reported engagement in protective behaviours and thus skewing intervention effects.

## Conclusion

Overall, our analyses provide a first, tentative indication that impulsivity may predict engagement in hygiene behaviours after the COVID-19 pandemic onset in a high risk, sub-Saharan African setting. Impulsivity therefore may require consideration within future pandemic planning. Interventions addressing behavioural factors may be of benefit and, health messaging may need targeting according to different population needs.

## Supplementary Information

**Additional file 1: Appendix 1.** Hygiene and social distancing behaviour items.

**Additional file 2: Appendix 2.** Correlations between the BART and individual hygiene and social distancing items.

**Additional file 3: Appendix 3.** Step-wise regression model predicting COVID social distancing behaviours during the past 7 days.

## Data Availability

The datasets generated and/or analyzed during the current study are not publicly available due to their sensitive nature but are available from the corresponding author on reasonable request.
