# Supplementary material for: Does pre-COVID impulsive behaviour predict adherence to hygiene and social distancing measures in youths following the COVID-19 pandemic onset? Evidence from a South African longitudinal study
Source: BMC Public Health. 2023 Mar 20;23:533. doi: 10.1186/s12889-023-15310-w (PMC10027426; doi:10.1186/s12889-023-15310-w)
Supplement: Supplementary file 1 — Additional file 1: Appendix 1. Hygiene and social distancing behaviour items. [file 12889_2023_15310_MOESM1_ESM.docx]

Appendix 1

*Hygiene and social distancing behaviour items*

| **Hygiene Behaviours** |
| --- |
| In the last 7 days, how often did you wash your hands with soap after being outside? |
| In the last 7 days, how often did you use hand sanitizer when in public/ after being in public? |
| In the last 7 days, how often did you wear a mask when you were in public? |
| In the last 7 days, how often did you cough/sneeze into your elbow? |
| **Social Distancing Behaviours** |
| In the last 7 days, how often did you stay at least 1-2 meters away from other people when in public? |
| In the last 7 days, how often did you avoid using public transportation? |
| In the last 7 days, did you avoid going to the grocery store or pharmacy? |
| In the last 7 days, did you avoid going into public spaces? |
| In the last 7 days, did you avoid going for walks in your neighbourhood? |

*Note.* Rating scale: 0 = Never, 1 = Rarely, 2 = Some of the time, 3 = Most of the time
