# Supplementary material for: Does pre-COVID impulsive behaviour predict adherence to hygiene and social distancing measures in youths following the COVID-19 pandemic onset? Evidence from a South African longitudinal study
Source: BMC Public Health. 2023 Mar 20;23:533. doi: 10.1186/s12889-023-15310-w (PMC10027426; doi:10.1186/s12889-023-15310-w)
Supplement: Supplementary file 2 — Additional file 2: Appendix 2. Correlations between the BART and individual hygiene and social distancing items. [file 12889_2023_15310_MOESM2_ESM.docx]

**Appendix 2**

*Correlations between the BART and individual hygiene and social distancing items*

|  | **1.** | **2.** | **3.** | **4** | **5.** | **6.** | **7.** | **8.** | **9.** |
| --- | --- | --- | --- | --- | --- | --- | --- | --- | --- |
| **1. BART** | **-** |  |  |  |  |  |  |  |  |
| **2. Hand sanitising** | -.07 | - |  |  |  |  |  |  |  |
| **3. Handwashing** | -.10 | .33 | - |  |  |  |  |  |  |
| **4. Wearing a mask** | .02 | .12 | .06 | - |  |  |  |  |  |
| **5. Cough/Sneeze into Elbow** | -.13 | .11 | .08 | .14* | - |  |  |  |  |
| **6. Keeping 1-2 m distance** | -.05 | .36* | .09 | .33* | .02 | - |  |  |  |
| **7. Avoiding public transport** | .05 | .07 | .01 | .11 | .03 | .13 | - |  |  |
| **8. Avoiding going to pharmacy/grocery store** | .01 | .20* | .04 | .01 | -.07 | .15* | .31* | - |  |
| **9. Avoiding public places** | -.07 | .14* | .06 | .05 | .06 | .23* | .16* | .40* | - |
| **10. Avoiding walks in neighbourhood** | -.07 | .24* | .20* | -.08 | .12 | .11 | -.02 | .20* | .38* |
