# Supplementary material for: Does pre-COVID impulsive behaviour predict adherence to hygiene and social distancing measures in youths following the COVID-19 pandemic onset? Evidence from a South African longitudinal study
Source: BMC Public Health. 2023 Mar 20;23:533. doi: 10.1186/s12889-023-15310-w (PMC10027426; doi:10.1186/s12889-023-15310-w)
Supplement: Supplementary file 3 — Additional file 3: Appendix 3. Step-wise regression model predicting COVID social distancing behaviours during the past 7 days. [file 12889_2023_15310_MOESM3_ESM.docx]

Appendix 3

*Step-wise regression model predicting COVID social distancing behaviours during the past 7 days*

|  | B (95% CI) | β | p |
| --- | --- | --- | --- |
| **Step 1** |  |  |  |
| BART pumps | -.01 (-.05; .04) | -.02 | .823 |
| *F, p, adjusted R^2^* | *0.05; .823; <.001* |  |  |
| **Step 2** |  |  |  |
| BART pumps | .01 (-.04; .06) | .02 | .804 |
| Sex (1= female) | 1.04 (.38; 1.71) | .21 | .002 |
| Age | .18 (-.36;.72) | .04 | .513 |
| Correct Class for Age | -.26 (-.91; .40) | -.05 | .440 |
| *F, p, adjusted R^2^* | *2.93;022;.053* |  |  |
| **Step 3** |  |  |  |
| BART pumps | .01 (-.04; .06) | .02 | .752 |
| Sex (1= female) | 1.06 (.39; 1.73) | .22 | .002 |
| Age | .19 (-.35; .73) | .05 | .492 |
| Correct Class for Age | -.24 (-.92; .43) | -.05 | .479 |
| Food Insecurity | -.03 (-.08; .03) | -.07 | .344 |
| Number of Household Members | -.04 (-.22; .13) | -.03 | .637 |
| Time to Level 1 Restrictions | -.01 (-.02; .01) | .08 | .233 |
| *F, p, adjusted R^2^* | *2.08; .047; .066* |  |  |
| **Step 4** |  |  |  |
| BART pumps | .01 (-.04; .06) | .04 | .615 |
| Sex (1= female) | 1.02 (.36; 1.69) | .21 | .003 |
| Age | .18 (-.36; .72) | .04 | .518 |
| Correct Class for Age | -.25 (-.92; .42) | -.05 | .462 |
| Food Insecurity | -.03 (-.08; .03) | -.06 | .369 |
| Number of Household Members | -.04 (-.22; .13) | -.04 | .612 |
| Time to Level 1 Restrictions | -.01 (-.02; .00) | -.09 | .212 |
| Intervention (1=yes) | .46 (-.19;1.12) | .10 | .166 |
| *F, p, adjusted R^2^* | *2.07;.040;.075* |  |  |

*Notes.* B = unstandardized regression coefficient, CI = confidence interval, β = standardized regression coefficient.
